# Dysregulation of liquid-liquid phase separation: from molecular mechanisms to targeted therapies in cardiovascular diseases

**DOI:** 10.1186/s12951-026-04627-4

**Published:** 2026-06-04

**Authors:** Xiaoyu Zhang, Xuejuan Ma, Ning Cao, Yuan Zhou, Zhigang Zhang, Runwei Ma

**Affiliations:** 1 Department of Cardiac Surgery, Fuwai Yunnan Hospital, Chinese Academy of Medical Sciences, Affiliated Cardiovascular Hospital of Kunming Medical University, Kunming, Yunnan Province China; 2 Department of Ultrasonography, The First Affiliated Hospital of Kunming Medical University, Kunming, Yunnan China; 3 Yunnan State Key Laboratory for Conservation and Utilization of Bioresources in Yunnan, School of Life Sciences, Yunnan University, Kunming, Yunnan Province China

**Keywords:** Liquid-liquid phase separation, Cardiovascular diseases, BC, M6A modification, Intrinsically disordered proteins

## Abstract

Liquid-liquid phase separation (LLPS), a fundamental biological process that drives the formation of membraneless organelles (MLOs) through non-covalent interactions, has emerged as a central regulatory mechanism in cellular physiology and stress responses. Increasing evidence links dysregulated LLPS to the pathogenesis of cardiovascular diseases (CVDs). However, the conserved molecular mechanisms underlying LLPS dysfunction across diverse CVDs, as well as the translation of LLPS-targeted strategies into clinical applications, remain poorly understood. In this review, we propose a novel hypothetical framework in which m6A modification-mediated RNA-intrinsically disordered protein (IDP) LLPS may serve as a conserved regulatory axis in CVDs, integrating the pathological mechanisms of multiple disease contexts. We further highlight that the irreversible transition of biomolecular condensates (BCs) from liquid to gel/solid states serves as a key driver of pathological irreversibility in end-stage CVDs, and demonstrate that m6A RNA modification and protein post-translational modifications (PTMs) act synergistically to regulate the RNA-IDP LLPS axis. Additionally, we summarize four emerging LLPS-targeted therapeutic strategies, including small-molecule modulators, biomaterial-based delivery systems, chiral nanomaterial interventions, and PTM-focused modulation. By connecting molecular mechanisms, disease relevance, and therapeutic potential, this review emphasizes the m6A-RNA-IDP axis and irreversible BC transitions as promising targets for broad-spectrum CVD intervention, providing a comprehensive framework to advance both basic research and translational nanobiotechnology applications.

**Graphical abstract:**

**Figure:**
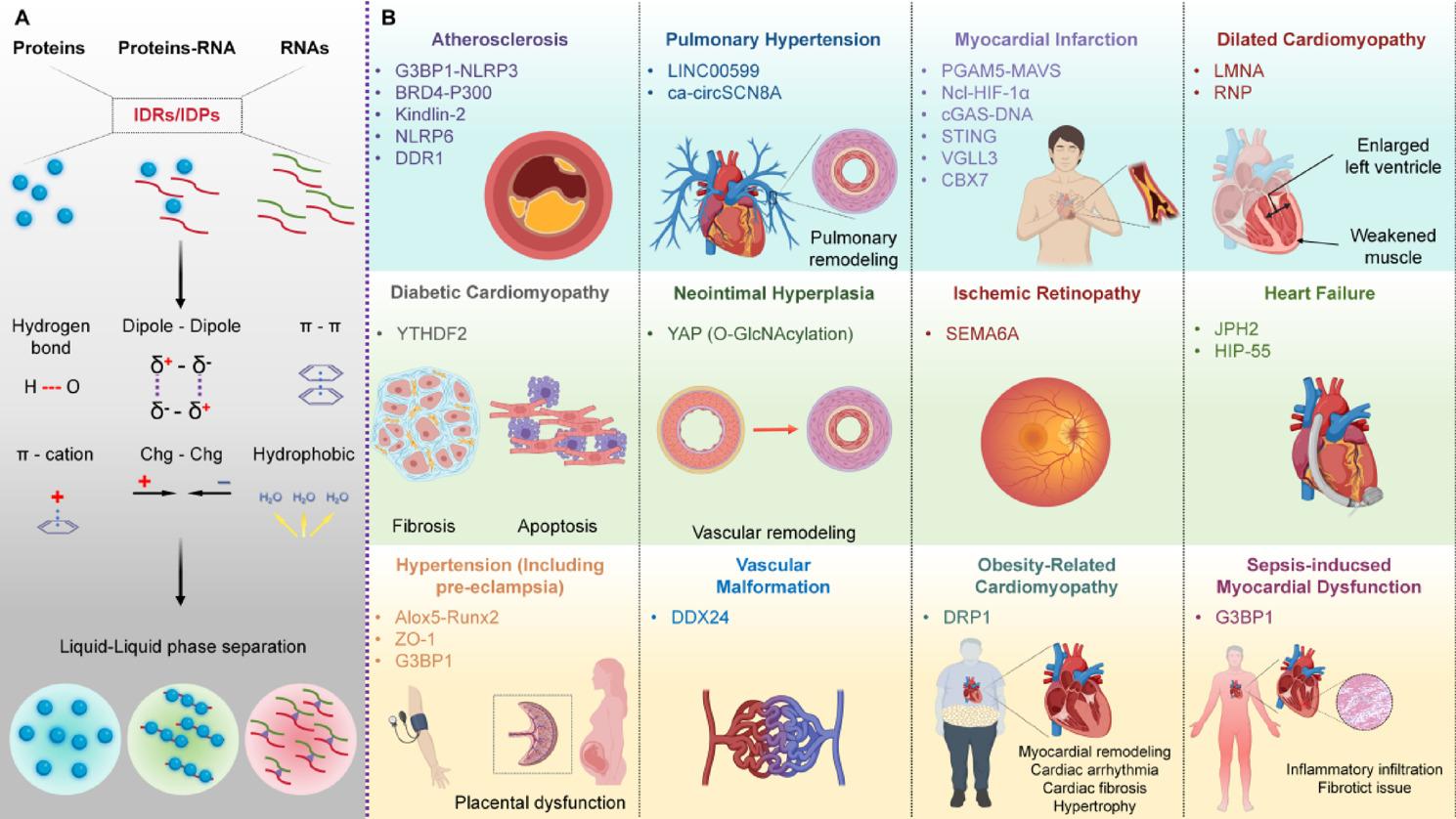

## Introduction

CVDs represent a significant public health challenge requiring urgent global attention [1]. It is projected that the global CVD-related mortality rate will increase by 73.4% between 2025 and 2050, with the annual number of deaths attributed to CVDs expected to rise to 35.6 million by 2050 [2]. Historically, research on the pathological mechanisms of CVDs has primarily focused on traditional areas such as abnormal signaling pathways and metabolic disorders; however, the emerging LLPS mechanism has provided a novel research dimension for deciphering the complex pathophysiological processes of CVDs [3].

LLPS is essentially a process wherein intracellular biomacromolecules (e.g., proteins, nucleic acids) spontaneously condense into droplet-like condensates (i.e., BCs) under specific physiological or pathological conditions [4, 5, 6]. These MLOs formed by LLPS possess both dynamism and functional specificity, enabling precise regulation of the local concentration of intracellular biomacromolecules while achieving fine spatiotemporal control of biochemical reactions [7, 8, 9]. Beyond participating in intracellular spatial compartmentalization and functional integration, LLPS also plays a key adaptive regulatory role in cellular responses to various stress stimuli, such as oxidative stress and viral infection [10]. Accumulating evidence has confirmed that dysregulated LLPS is closely associated with the development and progression of numerous major diseases, including neurodegenerative disorders and tumors [11–13, 14].

Although research on the LLPS mechanism in CVDs is relatively recent, existing evidence has clearly established its regulatory role in key pathological processes of CVDs, such as cardiomyocyte survival, maintenance of vascular endothelial function, and vascular smooth muscle cells (VSMCs) phenotypic transformation [9, 15]. Specifically, IDPs or proteins containing IDRs can drive LLPS through multivalent interactions [16, 17], and their dysfunction contributes to the development of CVDs [18, 19]. Furthermore, PTMs (e.g., arginine methylation, O-GlcNAcylation, phosphorylation) can indirectly modulate the activity of CVD-related signaling pathways by regulating the dynamic balance of LLPS [20, 21].

This review will elaborate on three aspects: the biological basis of LLPS, its mechanisms of action in common CVDs, and the progress of LLPS-targeted therapeutic strategies, aiming to provide references for the basic research and clinical translation of CVDs.

In recent years, a growing number of reviews have summarized the involvement of LLPS in tumors, inflammatory diseases, neurodegenerative disorders, and other conditions. However, existing reviews related to cardiovascular LLPS remain fragmented, limited to individual disease or signaling events, and lack systematic integration of conserved mechanisms and translational strategies. Notably, this article represents the first systematic and comprehensive review that focuses specifically on LLPS dysregulation across the full spectrum of CVDs.

Compared with previous reviews, the present study provides three key innovations and advancements: We propose a conserved m6A modification–mediated RNA–IDP LLPS regulatory axis that unifies pathological mechanisms across multiple CVDs. We identify irreversible liquid-to-gel/solid transition of BCs as a core driver underlying pathological irreversibility in end-stage CVDs. We systematically summarize four classes of emerging LLPS-targeted therapeutic strategies with clinical translational potential for CVDs.

This review therefore fills a critical gap in the field, establishes a unified mechanistic framework, and provides key insights for the development of broad-spectrum and stage-specific interventions for CVDs.

## Biological characteristics of LLPS

Cells are the fundamental structural and functional units of living organisms, and a key challenge is ensuring that intracellular components assemble in the correct spatiotemporal manner to execute precise physiological functions. To achieve this, cells have evolved two complementary types of organelles: membrane-bound organelles (e.g., mitochondria, nucleus, lysosomes), which are physically separated by membranes, and MLOs (e.g., nucleoli, P-bodies, Cajal bodies, dicing bodies, miRISC, and synaptic cytoskeleton) formed through dynamic biomolecular assembly [22–24, 25]. While membrane-bound organelles have been extensively studied, with well-established structural, mechanistic, and regulatory frameworks, key questions about MLOs, such as their formation pathways and fundamental physicochemical properties, remain incompletely understood [26].

### Characteristics of LLPS

To ensure clarity and consistency, key terms are defined upfront:

- LLPS: A specific reversible thermodynamic process wherein soluble biomolecules demix into liquid-like dense phases and dilute phases via weak non-covalent interactions.
- Phase separation (PS): A broad biological process encompassing the partitioning of biomolecules into distinct phases, including liquid–liquid, liquid–gel, and liquid–solid transitions.
- BCs: Dynamic, membraneless organelles or compartments formed primarily by LLPS, which execute spatiotemporal regulation of cellular activities.

LLPS is the spontaneous demixing of soluble biomolecules into a concentrated phase (condensates) and a dilute phase (cytoplasm or nucleoplasm) via non-covalent interactions, including hydrogen bonding, π-cation, π-π, cation-cation, hydrophobic, and dipole–dipole interactions [8, 24–28, 29]. Condensates display diverse material states, from liquid-like to viscoelastic or gel-like, depending on molecular composition, droplet maturation, and the system’s position within the two-phase regime [30]. FRAP is widely used to assess molecular exchange, e.g., HIP-55 condensates recover over 60% of fluorescence [31], but recovery depends on droplet size, mobility, and bleached region, so FRAP alone is not definitive for LLPS [32].

LLPS enriches specific molecules to form BCs. Stress granules (SGs) are composed of key components including G3BP1, mRNA, and translation factors [33, 34], whereas nuclear condensates mainly contain transcription factors (TDP-43, Runx2, FOXM1, FUS) and coactivators (P300) [35–39, 40]. Through specific intermolecular interactions in a membrane-free context, BCs provide precise spatial compartmentalization, dynamic regulation, and error avoidance [41], supporting signal sensing [42], reaction modulation [43, 44], buffering [45], localization [46], mechanical force generation [47, 48], and selective filtering [49], thereby impacting cellular physiology, stress adaptation, and disease.

**Fig. 1:**
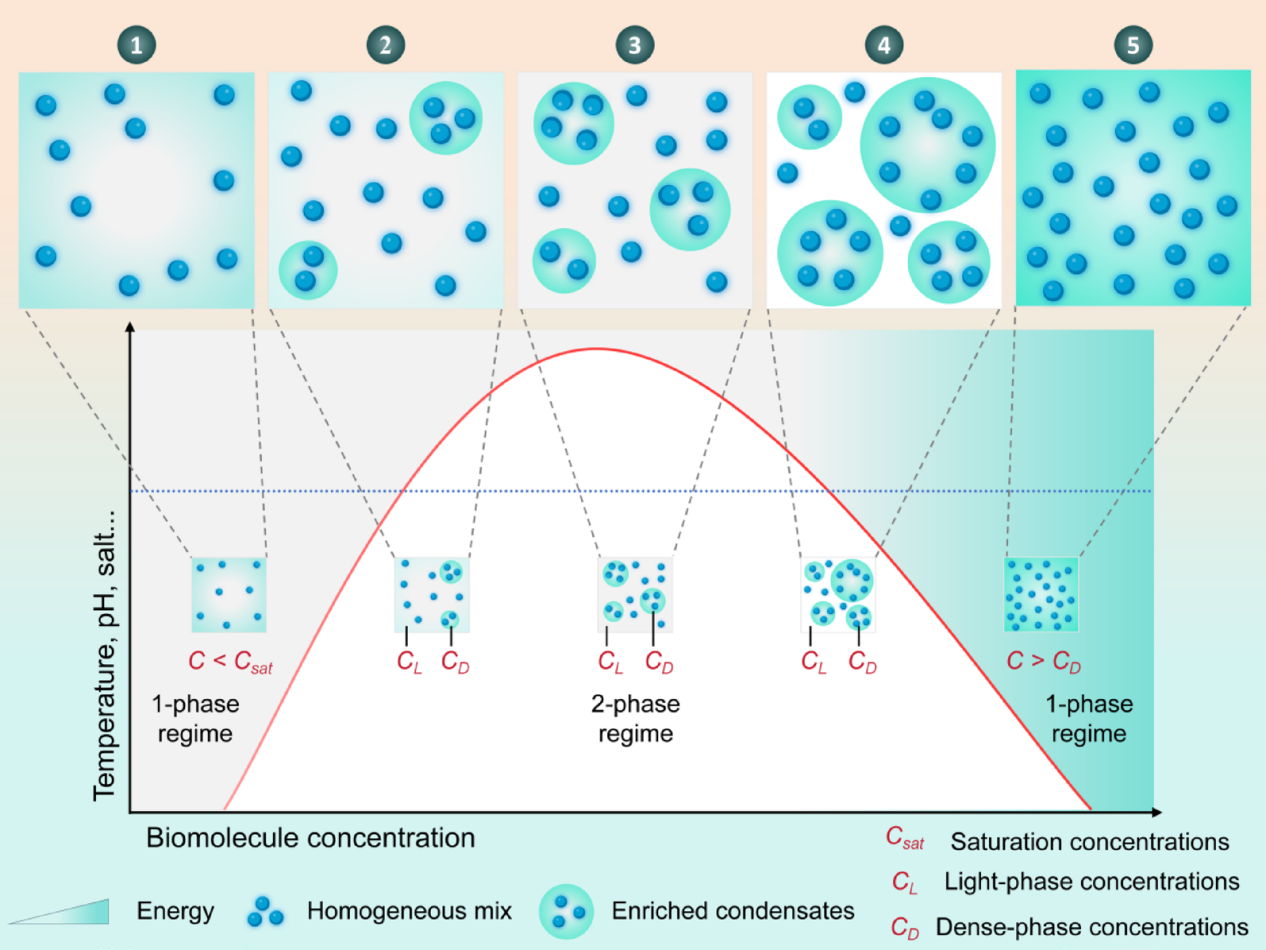
Phase diagram and thermodynamic characteristics of LLPS. The x-axis represents the concentration of biomolecules. The y-axis denotes environmental regulatory factors (including temperature, pH value, ionic strength.), which can affect LLPS equilibrium by altering the charge state and interaction strength of biomolecules. *C*_*sat*_: Critical concentration for LLPS initiation; *C*_*L*_: Equilibrium concentration of biomolecules in the dilute phase when two phases coexist; *C*_*D*_: Equilibrium concentration of biomolecules in the condensed phase during two-phase coexistence. 1-phase regime (①): When the biomolecule concentration is lower than *C*_*L*_, the system exists in a homogeneous single-phase state. At this point, biomolecules are uniformly dispersed in the solution, with no obvious condensate formation, corresponding to the energy and state characteristics of the Homogeneous mix in the figure. 2-phase regime (②-④): When the biomolecule concentration is between*C*_*L*_ and* C*_*D*_and environmental factors are within an appropriate range, LLPS occurs, and the system enters a two-phase coexistence state. Biomolecules spontaneously separate into a molecule-poor dilute phase and a molecule-enriched condensed phase (Enriched condensates), maintaining dynamic equilibrium. With the increase in total biomolecule concentration, the volume fraction of the condensed phase gradually increases while that of the dilute phase decreases accordingly; however, the biomolecule concentrations *C*_*L*_and *C*_*D*_ in each phase remain constant. 1-phase regime (⑤): When the biomolecule concentration exceeds *C*_*D*_, the system returns to a single-phase state. At this time, biomolecules form a homogeneous high-concentration system, and PS no longer occurs, with its energy state significantly differing from that of the low-concentration single-phase region on the left. Thermodynamic characteristics [59]: The transition from ① to ④ proceeds without energy consumption (an entropy-increasing process); the transition from ④ to ⑤ requires energy input (an entropy-decreasing process).

LLPS is highly sensitive to environmental cues (Fig. 1), including temperature, pH, salt concentration, molecular crowding, and PTMs. High osmotic concentrations can inhibit IDP-mediated condensate formation, whereas arginine methylation reduces Kindlin-2 LLPS [50]. Microenvironmental pH alters protein/RNA charge and interactions, affects solubility, and acts as a stress signal [22, 34, 51–57, 58].

### Driving molecules and regulatory mechanisms of LLPS

IDRs, despite lacking stable tertiary structures, serve as versatile RNA-binding modules. Enriched in positively charged or aromatic residues, they engage RNA via electrostatic and stacking interactions with low sequence specificity, often coexisting with canonical RNA-binding domains and contributing to both RNA recognition and the assembly of membraneless organelles through PS [60]. IDPs and IDRs are central drivers of LLPS, forming reversible condensates via low-complexity sequences, repetitive motifs, and multivalent weak interactions [18, 61]. IDR charge patterns serve as molecular codes [62], directing selective biomolecule enrichment: activators with charged blocks preferentially localize to transcriptional condensates, whereas repressors with uniform charges are excluded, and modulation of these patterns reshapes condensate composition. Notably, DDR1 undergoes LLPS in VSMCs under mechanical stimulation, co-condensing with LATS1 to regulate Hippo-YAP signaling [19, 63, 64].

BCs partitions cellular contents and plays essential roles in stress responses, homeostasis, development, and disease. Many nuclear and cytoplasmic condensates are enriched in RNA and RNA binding proteins (RBPs), which undergo LLPS [65]. While the role of RBPs has been extensively studied, RNA contributions to LLPS remain less explored. Nucleic acids, particularly RNA, modulate LLPS in a concentration-dependent manner: low RNA/protein ratios promote, whereas high ratios inhibit, prion-like RBP condensation [45, 66, 67, 68]. m6A-modified RNAs interact with YTHDF2, whose N-terminal low-complexity domain facilitates PS, implicating RNA-mediated LLPS in cardiovascular pathogenesis [69, 70, 71]. Proteins such as G3BP1 initiate RNA-dependent SG assembly via IDR interactions, functioning as hub proteins with multiple interaction sites [62], regulated by PTMs such as JMJD6-mediated arginine demethylation [72, 73].

LLPS also underpins transcriptional regulation: RNA polymerase Ⅱ CTD condensates coordinate transcription initiation, with phosphorylation triggering dissolution [74], and pre-initiation complex components exploit IDR interactions to enhance assembly [75].

LLPS dynamics are tightly regulated by PTMs [76], enzymatic activity, environmental factors, and mechanical forces. PTMs including SUMOylation [77], acetylation [78], and ubiquitination [79], together with enzymes, collaboratively control condensate composition and behavior [62]: PRMT5-mediated methylation of Kindlin-2 inhibits LLPS and endothelial adhesion [50]; O-GlcNAcylation enhances YAP multivalency, promoting co-condensation with STAT3 [21] and driving CEP44 droplet fusion for centriole duplication [80]; PRMT8 and TRIM21 modulate G3BP1-mediated SGs [81, 82]; phosphorylation of FUS disrupts LLPS, reducing aggregation and cytotoxicity [83]. PTMs can act synergistically, as shown for CEP44, where O-GlcNAcylation and phosphorylation co-regulate structure and localization [80]. Enzymes further shape LLPS by catalyzing substrate transformations, controlling material flow or degradation, and remodeling condensate subcompartments, acting as both “catalysts” and “architects.”

Environmental and mechanical cues modulate intermolecular interactions and condensate organization. Hypoxia-induced lactate promotes AARS1-mediated p53 lactylation, suppressing p53 LLPS and DNA binding [84]. Cells buffer component distribution between dilute and condensed phases [62], while physiological shear stress preserves Kindlin-2 LLPS and barrier integrity, and pathological oscillatory stress induces arginine methylation, suppressing LLPS [50]. Mechanical signals also govern SG assembly via Filamin A-G3BP1 interactions, and modulation of hub proteins such as G3BP1 alters SG-P-body dynamics [62], impacting endothelial stress responses and contributing to vascular pathologies [85].

**Fig. 2:**
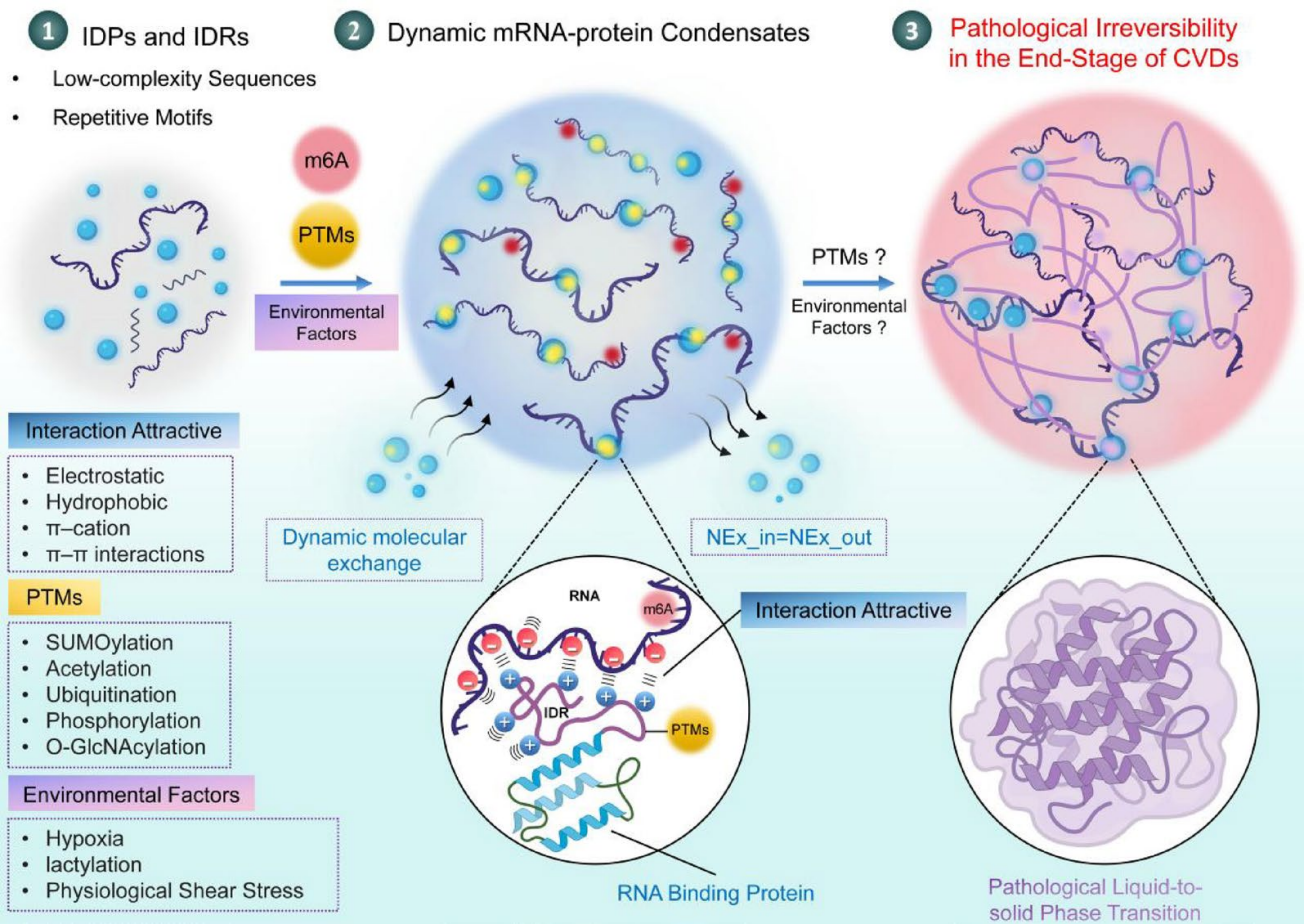
Dynamic mRNA-protein condensates and pathological liquid-to-solid phase transition in CVDs. (①) IDPs and IDRs drive condensate formation via low-complexity sequences and repetitive motifs. Attractive interactions, including electrostatic, hydrophobic, π-cation, and π-π contacts, promote assembly. (②) Dynamic mRNA-protein condensates exhibit reversible molecular exchange (NEx_in = NEx_out) and selective recruitment of RNA and RNA-binding proteins, modulated by PTMs (SUMOylation, acetylation, ubiquitination, phosphorylation, O-GlcNAcylation), RNA modifications (e.g., m6A), and environmental factors (hypoxia, lactylation, shear stress). (③) Pathological irreversibility in the end stage of CVDs may arise from dysregulated PTMs and environmental stresses, triggering liquid-to-solid phase transitions and aberrant protein aggregation

Collectively, LLPS formation and homeostasis are orchestrated by a PTM-centered network integrating RNA m6A and protein modifications, environmental cues, and mechanical forces. Under this regulation, IDPs/IDRs form stable RNA-IDP condensates, and dysregulation of the PTM network is closely associated with the onset and progression of CVDs (Fig. 2).

## Mechanisms underlying the roles of LLPS in common CVDs

### Myocardial infarction (MI)

The core pathological processes of MI include myocardial ischemia reperfusion injury (MIRI), inflammatory response, and myocardial fibrosis. LLPS participates in disease progression by regulating the following:

#### Regulation of cardiomyocyte pyroptosis

During MIRI, the expression of the E3 ubiquitin ligase MARCH2 is upregulated, which binds to phosphoglycerate mutase 5 (PGAM5) and promotes its K48-linked ubiquitination and degradation, thereby reducing the LLPS-mediated co-condensation of PGAM5 and mitochondrial antiviral signaling protein (MAVS). Given that PGAM5-MAVS condensates can recruit and activate the NLRP3 inflammasome, MARCH2 ultimately inhibits the pyroptosis pathway and alleviates MIRI [86].

In another mechanism, ischemia-induced upregulation of SUMO1 promotes SUMOylation of hypoxia-inducible factor HIF-1α at K477. Meanwhile, the nucleolar protein Ncl stabilizes HIF-1α mRNA through LLPS, increasing its protein level. Elevated HIF-1α further induces NLRP3 inflammasome expression and the production of various inflammatory molecules, thereby exacerbating myocardial pyroptosis. These findings suggest that both the Ncl-LLPS-HIF-1α and SUMO1-HIF-1α axes may serve as essential targets for intervention in pyroptosis-related injury [87].

In addition, age-related functional drift of CBX7 is also involved in pyroptosis regulation. In aged hearts, CBX7 forms LLPS condensates with the copper transporter ATP7A, retaining ATP7A intracellularly and reducing its transmembrane transport capacity, thereby inducing cuproptosis. In contrast, CBX7 primarily promotes cardiomyocyte proliferation in young hearts [88].

#### Promotion of cardiac fibrosis

After MI, increased matrix stiffness induces the nuclear translocation of the VGLL3 protein in cardiac myofibroblasts via the integrin β1-Rho-actin pathway. VGLL3 undergoes LLPS via its low-complexity domain (LCD) and co-condenses with the EWSR1 protein to form intranuclear condensates, which, in turn, inhibit miR-29b expression (a molecule that targets collagen mRNA), ultimately promoting collagen deposition and cardiac fibrosis [89].

### Hypertension

The pathological core of hypertension includes vascular remodeling, endothelial dysfunction, and target organ damage. LLPS participates in disease mechanisms by regulating transcription factor activity and ciliary function:

#### Regulation of VSMCs hypertrophy

In the hypertensive state, arachidonate 5-lipoxygenase (ALOX5) is upregulated in cardiomyocytes. Its PEST domain binds to the PTS domain of Runx2, promoting the formation of intranuclear LLPS of Runx2 in an enzyme activity-independent manner. Runx2 condensates enhance transcriptional activity of the EGFR gene, leading to abnormal VSMCs proliferation and cardiac hypertrophy. Targeting Alox5 can interfere with Runx2 LLPS and alleviate hypertension-induced ventricular remodeling [37].

#### Ciliary dysfunction in preeclampsia (gestational hypertension)

Patients with preeclampsia exhibit reduced quantity and impaired function of primary cilia in placental trophoblasts, a mechanism associated with LLPS-mediated ciliary signaling disorders. Imbalanced dietary lipids reduce membrane cholesterol availability, inhibit the LLPS of molecules related to the Hedgehog/Wnt signaling pathway in cilia, and cause placental angiogenesis disorders. Restoring LLPS function can enhance trophoblast invasiveness and alleviate placental insufficiency in preeclampsia [90].

#### Vascular barrier injury mediated by tight junction dysfunction

ZO-1 PS is essential for the proper assembly of tight junction (TJ) complexes, the establishment and maintenance of apical–basal polarity in epithelial and endothelial cells, and the preservation of tissue paracellular permeability homeostasis [91, 92]. Dysregulation of paracellular permeability and associated ion transport abnormalities are closely linked to the development and progression of cardiovascular and metabolic diseases, including hypertension [93, 94]. Understanding how aberrant ZO-1 PS disrupts TJ assembly and maintenance, and elucidating its underlying molecular regulatory network, could provide new mechanistic insights into TJ-related disorders such as hypertension and hereditary barrier defects, while offering a theoretical foundation for developing therapeutic strategies targeting ZO-1 PS and TJ function.

### AS

The key pathological processes of AS include foam cell formation [95], endothelial barrier disruption, and inflammatory response [96]. LLPS participates in disease progression by regulating lipid metabolism and endothelial adhesion function:

#### Regulation of foam cell inflammatory response

Oxidized low-density lipoprotein (ox-LDL) induces autophagy defects in macrophages, leading to cytoplasmic retention of transcription factor EB (TFEB) and subsequent activation of the P300-BRD4 axis. BRD4 can form super-enhancer condensates through LLPS [97, 98], which are enriched in the promoter regions of inflammatory genes (e.g., IL-6, TNF-α), enhancing inflammatory responses and promoting atherosclerotic plaque formation [99].

Furthermore, other metabolic and stress factors can further strengthen the inflammatory phenotype of foam cells through autophagy-related pathways. Studies have demonstrated that glycaemic variability can affect NIH and plaque stability after coronary stent implantation via the autophagy-mediated G3BP1/NLRP3 inflammasome signaling axis, acting as a tunable switch that triggers PS for SG assembly [34]. Suppressing G3BP1-driven PS or disrupting its interaction with NLRP3 directly attenuates NLRP3 inflammasome activation, highlighting the mechanistic role of G3BP1 in inflammasome regulation [100].

NOD-like receptor family pyrin domain-containing 6 (NLRP6) is an essential member of the NLR family, which was previously considered to affect host susceptibility to infections and tumors primarily by regulating microbiota composition and local inflammatory responses in intestinal epithelial cells [101, 102]. Recent studies have found that NLRP6 can drive LLPS via its lysine-rich region within the IDR [103], thereby promoting inflammasome assembly upon viral or intestinal flora stimulation. Its PYD domain further self-assembles into fibers and recruits ASC, leading to droplet solidification and prolonged inflammatory signaling. Dysregulation of NLRP6 LLPS may accelerate the progression of CVDs such as AS by amplifying inflammatory responses [104].

#### Regulation of signal transduction

In the pathological progression of AS, in addition to regulating gene expression, PS also plays a crucial regulatory role in signal transduction pathways. Studies by Liu et al. [105] have confirmed that blood flow shear stress can induce conformational rearrangement of (DDR1 in endothelial cells, thereby promoting its oligomerization and initiating PS, ultimately forming droplet-like BCs. Oligomerized DDR1 undergoes autophosphorylation and forms a co-condensate with tyrosine 3-monooxygenase/tryptophan 5-monooxygenase activation protein ε (YWHAE). This complex can block the phosphorylation of YAP, promoting its translocation from the cytoplasm to the nucleus, which, in turn, activates the expression of pro-inflammatory and proliferation-related target genes, representing a potential molecular mechanism that initiates and drives the progression of AS.

Furthermore, in VSMCs, DDR1 can be co-induced to undergo PS by mechanical and chemical signals from the extracellular matrix. The formed condensates can co-condense with large tumor suppressor kinase 1 (LATS1), a key molecule in the Hippo pathway. The formation of DDR1-LATS1 condensates inhibits LATS1 phosphorylation and inactivates LATS1, thereby blocking downstream YAP phosphorylation. This not only induces phenotypic transformation of vascular VSMCs, leading to their excessive proliferation and migration, but also promotes collagen deposition, further exacerbating extracellular matrix sclerosis. This process forms a positive feedback loop for DDR1-YAP activation, ultimately accelerating the pathological progression of AS [19].

#### Maintenance of vascular endothelial stability

Kindlin-2 is a core component of endothelial adherens junctions and focal adhesions. Under pulsatile shear stress, Kindlin-2 mediates LLPS via the R290 residue, promoting focal adhesion assembly and maturation. In contrast, oscillatory shear stress induces PRMT5 to catalyze arginine methylation of Kindlin-2 at R290, thereby inhibiting its LLPS activity, leading to increased endothelial cell permeability and enhanced monocyte adhesion. Inhibiting arginine methylation can restore the LLPS activity of Kindlin-2 and alleviate atherosclerotic lesions [50].

### Pulmonary hypertension (PH)

The core pathological feature of PH is pulmonary vascular remodeling. LLPS participates in disease mechanisms by regulating pulmonary artery smooth muscle cell (PASMC) proliferation and ferroptosis.

#### Regulation of PASMC proliferation

Hypoxia induces upregulated expression of LINC00599 in the medial layer of pulmonary arteries. LINC00599 binds to G3BP1 and MYH9 proteins via m6A modification, driving LLPS to form SG-like condensates. These condensates can activate cell cycle-related signaling pathways and promote PASMC proliferation. Lentivirus-mediated silencing of LINC00599 can inhibit its LLPS activity and reverse hypoxia-induced pulmonary vascular remodeling in PH [70].

#### Regulation of PASMC ferroptosis

The expression of ca-circSCN8A is upregulated in pulmonary artery tissues of PH patients. ca-circSCN8A can recruit EP300 to promote lactylation modification of the FUS protein, thereby inducing LLPS of the ca-circSCN8A/FUS/EP300 complex. This condensate stabilizes the R-loop formed between ca-circSCN8A and the promoter of the ferroptosis-related gene SLC7A11, thereby inhibiting SLC7A11 transcription, reducing glutathione synthesis, and enhancing oxidative stress, ultimately inducing PASMC ferroptosis. Targeting ca-circSCN8A can disrupt LLPS-mediated R-loop formation and alleviate PH progression [106].

#### Regulation of PASMC stiffening

In addition to the aforementioned regulatory mechanisms underlying proliferation and ferroptosis, stiffening of the vascular extracellular matrix represents an early and ubiquitous driver in the development and progression of PH. Liu et al. experimentally demonstrated that the receptor tyrosine kinase Eph receptor B4 (EphB4) is a key molecule mediating the aberrant responses of PASMCs to matrix stiffening. Further investigations revealed that matrix rigidity promotes conformational extension of the IDR at the C-terminus of EphB4, thereby inducing its LLPS to form BCs. These condensates specifically sequester the regulatory proteins of YAP, primarily ANXA2 and YWHA, thus disrupting the cytoplasmic retention of YAP, facilitating its nuclear translocation, and ultimately driving abnormal proliferation of PASMCs [107].

### Diabetic cardiomyopathy (DbCM)

In DbCM, a high-glucose environment induces downregulated expression of Alkbh5 in cardiac fibroblasts, leading to increased m6A modification levels in the 3’ untranslated region (3’UTR) of Notch1 mRNA. This elevation enhances the LLPS activity of the YTHDF2 protein [108], thereby promoting the recognition of Notch1 mRNA by YTHDF2, which, in turn, inhibits the Notch1 signaling pathway, ultimately leading to abnormal mitochondrial fission and cardiac fibrosis. Experimental evidence has demonstrated that overexpression of Alkbh5 can reduce the m6A level of Notch1 mRNA, inhibit YTHDF2-mediated LLPS, and alleviate diabetes-induced myocardial fibrosis [109]. Therefore, as the most common modification in mRNA, m6A not only regulates the fate of mRNA itself but also significantly influences the LLPS activity of its binding proteins [71].

Additional studies have confirmed that mRNAs containing multiple m6A residues can serve as multivalent scaffolds for the m6A reader protein YTHDF2. The low-complexity domains of YTHDF2 mediate its LLPS, facilitating the assembly of dynamic mRNA-protein condensates [110]. Notably, the mechanism by which YTHDF family proteins enhance LLPS and regulate target RNA functions through m6A-modified RNA is universal, with similar phenomena observed in other disease models [111], suggesting that this regulatory pattern may represent a conserved cross-disease molecular mechanism.

### Heart failure (HF)

**Fig. 3:**
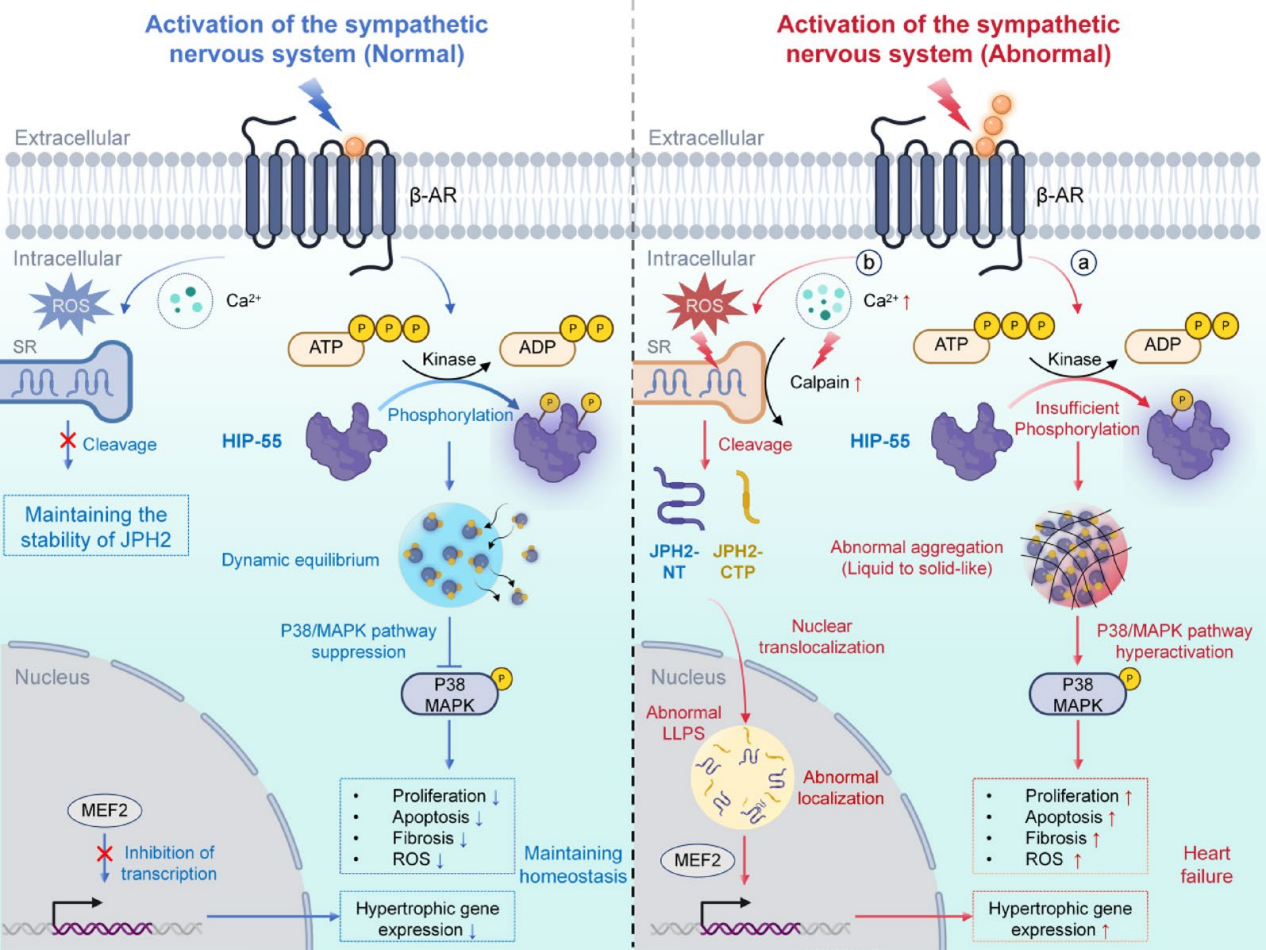
Effects of sympathetic nervous system activation status on LLPS and cardiac function regulatory pathways. Left panel (Blue arrows): Normal activation of the sympathetic nervous system. HIP−55 is sufficiently phosphorylated, maintaining normal LLPS activity; JPH2 is not cleaved and sustains structural stability through dynamic equilibrium, ensuring myocardial excitation-contraction coupling. Intracellular Ca^2+^ concentration and reactive ROS levels are in physiological homeostasis, with standard ATP/ADP conversion and orderly regulation of protein kinase A (PKA) activity. This prevents excessive activation of the P38/MAPK pathway, while safeguarding the normal transcriptional function of the transcription factor MEF2, thereby maintaining the homeostasis of cardiomyocyte gene expression. Right panel (Red arrows): Abnormal activation of the sympathetic nervous system. Insufficient phosphorylation of HIP−55 leads to abnormal LLPS function, preventing the formation of functional condensates and instead forming solid-like aggregates. Under abnormal conditions, JPH2 is cleaved into the JPH2-CTP fragment, which undergoes aberrant nuclear translocation and loses normal function. Intracellular Ca^2+^ concentration increases, ROS accumulates, energy metabolism (ATP/ADP conversion) is disrupted, and kinase regulation is imbalanced—resulting in excessive activation of the P38/MAPK pathway and triggering downstream pro-apoptotic and pro-fibrotic signaling cascades. The transcriptional function of MEF2 is significantly inhibited, leading to dysregulation of cardiomyocyte homeostatic gene expression

Sympathetic overactivation is a key driver of HF progression and can reduce the phosphorylation levels of the HIP-55 protein at S269 and T291. Defective phosphorylation of HIP-55 impairs its LLPS capacity, leading to insoluble aggregates that, in turn, abrogate its inhibitory effect on the β-adrenergic receptor-mediated P38/MAPK pathway, exacerbating cardiomyocyte apoptosis and cardiac dysfunction. Cardiac-specific overexpression of wild-type HIP-55 can restore its LLPS activity, inhibit excessive activation of the P38/MAPK pathway, and alleviate HF symptoms [31]. It is worth noting that sympathetic activation in HF is often accompanied by enhanced oxidative stress and calpain-2 activation [112], which can also affect the function of JPH2, another key cardiac protein. Oxidative stress can promote the translocation of full-length JPH2 into the nucleus via a bipartite nuclear localization signal to form PS nuclear droplets, whose dysfunction may exacerbate myocardial remodeling [113, 114]. Additionally, calpain-2 can cleave JPH2 to generate the C-terminal fragment JPH2-CTP, which promotes adverse myocardial remodeling following nuclear localization [112, 115]. Therefore, as a core protein maintaining myocardial excitation-contraction coupling, abnormal LLPS and JPH2 loss-of-function may synergize with HIP-55 deficiency to accelerate cardiac function deterioration (Fig. 3).

### NIH

Following vascular injury, the level of O-GlcNAcylation modification of the YAP protein in VSMCs is elevated. This modification enhances YAP’s multivalency, promoting LLPS with STAT3 and nuclear translocation. Intranuclear YAP-STAT3 condensates can activate transcription of target genes, such as CYR61 and PCNA, driving abnormal proliferation and migration of VSMCs. The small molecule chrysophanol can inhibit the O-GlcNAcylation modification of YAP, reduce its LLPS activity, and thereby suppress the progression of NIH [21].

### Dilated cardiomyopathy (DCM)

DCM is one of the common diseases causing HF, arrhythmia, and sudden cardiac death [116]. Its etiology exhibits significant genetic heterogeneity [117], and more than 50 related pathogenic genes have been identified, among which mutations in LMNA and RBM20 are closely associated with disease onset [118–120, 121]. Dysregulated LLPS plays a key role in the pathogenesis of DCM, and relevant studies have further revealed the core mechanisms by which these pathogenic genes mediate the disease through LLPS disorders.

Dysfunction of lamin A/C is closely linked to DCM. A recent study by Nath et al. found that high-frequency lamin A mutants E161K and K97E in DCM disrupt the peripheral laminar structure, leading to heterochromatin disorganization and the formation of lamin A aggregates. Simulation experiments verified that disruption of lamin-nuclear membrane-chromatin interactions can recapitulate the phenotype, and three-dimensional FISH confirmed abnormal HC localization and lamin dynamics of the mutants. The mesoscale characteristics of the aggregates suggest LLPS features [122].

In addition, dysregulation of ribonucleoprotein (RNP) granules is involved in DCM pathogenesis. Schneider et al. observed abnormal accumulation of sarcoplasmic RNP granules in gene-edited pigs with DCM, a phenomenon that was verified in myocardial tissue from patients and in reprogrammed cells. Dysregulated RBM20 RNP granules exhibit liquid properties, dock along the cytoskeleton, and promote PS, which is associated with myocardial pathology and HF ^*123*^. Kornienko et al. further found that mutations in the RS domain of RBM20 disrupt its interaction with transportin-3 (TNPO3), leading to cytoplasmic mislocalization and the formation of toxic granules. In contrast, nuclear relocalization can restore splicing function and dissolve the granules [124].

### Obesity-related cardiomyopathy (ORCM)

ORCM is a cardiac syndrome caused by obesity, characterized by hypertrophy and diastolic dysfunction [125, 126]. Mitophagy plays an essential role in the development of obesity-induced cardiomyopathy [126], [127].

#### Potential Link between LLPS and ORCM

Dynamin-related protein 1 (DRP1) serves as a core mechanical enzyme in mitochondrial fission. The variable domain (VD) of DRP1 exhibits typical intrinsically disordered features and can undergo LLPS to form liquid-like condensates under intracellular crowding or specific cosolute induction [128]. This process enhances the binding of DRP1 to cardiolipin and promotes the assembly of mitochondrial fission machinery, suggesting that LLPS may act as a structural basis for DRP1-mediated mitochondrial dynamics [129].

Although numerous studies have reported targeting DRP1-mediated mitophagy in CVDs [130, 131, 132], studies that directly link DRP1-mediated LLPS to cardiovascular pathological changes remain scarce, and current evidence is mostly derived from in vitro biochemical characterization rather than in vivo ORCM models. Nevertheless, existing findings imply a potential regulatory axis: in high-fat diet (HFD)-induced ORCM, dysregulation of DRP1 LLPS may disturb mitophagy and mitochondrial quality control, thereby contributing to myocardial dysfunction [133]. DRP1 phosphorylation at Ser616, mitochondrial localization, and interactions with Rab9 and Fis1 are closely associated with PS-mediated dynamic assembly, but direct in vivo evidence supporting a pathological role of DRP1 LLPS in ORCM is still lacking. Accordingly, mature therapeutic strategies targeting the DRP1 LLPS axis in CVDs have not yet been developed.

#### Proposed experimental strategies to validate the DRP1 LLPS axis in ORCM

To verify the functional role of DRP1 LLPS in the pathogenesis of ORCM, the following experimental schemes are suggested:

- In vitro PS verification: Recombinantly express wild-type DRP1 and IDR-truncated/VD-mutated DRP1, and compare their LLPS behaviors under physiological or high-fat–mimicking conditions.
- Cellular functional validation: Use DRP1 LLPS deficient mutants in cardiomyocytes or cardiac fibroblasts treated with palmitate to mimic high-glucose and high-lipid-induced cardiomyocyte injury, and detect changes in mitochondrial fission, mitophagy, apoptosis, and hypertrophy.
- In vivo animal model validation: Construct cardiac-specific DRP1 IDR-mutant knock-in mice, establish HFD-induced ORCM models, and evaluate cardiac function, mitochondrial homeostasis, and myocardial remodeling.
- Pharmacological intervention: Use LLPS-modulating small molecules to target DRP1 condensates and assess whether cardiac dysfunction can be alleviated in ORCM models.

Collectively, DRP1 LLPS represents a potential but not fully validated mechanism underlying ORCM. Further targeted experiments are urgently needed to confirm its pathological contribution and translational value.

### Sepsis-induced myocardial dysfunction

Sepsis-induced myocardial dysfunction is closely associated with SGs-LLPS dysfunction. Lipopolysaccharide can activate G3BP1-mediated SG formation in cardiomyocytes, which reduces TNF-α expression, improves mitochondrial membrane potential, and alleviates cardiomyocyte contractile dysfunction. Overexpression of G3BP1 can enhance SG LLPS activity, further protecting cardiomyocytes from LPS-induced damage, suggesting that targeting G3BP1-LLPS may represent a promising therapeutic strategy for sepsis-induced myocardial injury [134].

### LLPS dysregulation mechanisms in other vascular-related diseases

In pathological angiogenesis diseases such as ischemic retinopathy, LLPS dysregulation is a core pathological driver of disease progression [135]. Its mechanism shares commonalities with LLPS abnormalities in HF: the transmembrane protein SEMA6A forms phase separated condensates via its C-terminal IDR, recruits RHOA and P300, and promotes the phosphorylation of P300, thereby inducing histone H3K9la/H3K18la modifications. These modifications activate PRMT5 transcription, enhance endothelial cell glycolysis and lactate accumulation, and form a positive feedback loop of lactate-SEMA6A LLPS-histone lactylation-PRMT5, accelerating the abnormal proliferation of neovascularization. Endothelial-specific knockout of SEMA6A or PRMT5 can reduce retinal neovascularization and improve ischemia, suggesting that this metabolism-epigenetics-LLPS axis is conserved in vascular diseases. Targeting SEMA6A IDR, P300, or PRMT5 may represent a cross-disease therapeutic direction.

Dysregulated LLPS control of nucleolar homeostasis is also involved in vascular malformations [136]. The nucleolar protein DDX24 (DEAD-box helicase) forms LLPS condensates via its IDR, binds to the nucleolar scaffold protein NPM1 in a client capacity, and modulates NPM1’s PS. In patients with multiple congenital venous and lymphatic malformations (MOVLD), the E271K mutation of DDX24 (located in the IDR) reduces its partitioning efficiency into nucleolar condensates, leading to decreased NPM1 mobility and abnormal nucleolar morphology. DDX24 mutation or knockdown also disrupts NPM1 LLPS function, inhibits pre-ribosomal RNA (47s rRNA) processing and 5.8s rRNA nuclear export, causes ribosome biogenesis defects in endothelial cells, and promotes vascular malformations. Overexpression of wild-type DDX24 can restore normal NPM1 PS and nucleolar homeostasis, reversing the abnormal endothelial cell phenotype. This study establishes, for the first time, a direct link between nucleolar LLPS dysregulation and vascular malformations, suggesting that nucleolar protein PS regulation may represent a common pathological mechanism in vascular diseases.

This section systematically elaborates on the roles of LLPS in regulating core pathological processes, including cell pyroptosis, fibrosis, and vascular remodeling. In addition, LLPS participates in diseases, including NIH and dilated cardiomyopathy, by regulating YAP-STAT3 co-condensation and LMNA/RBM20 aggregates. Moreover, conserved mechanisms such as the metabolism-epigenetics-LLPS axis exist in ischemic retinopathy and vascular malformations, confirming that LLPS may serve as a key molecular mechanism linking the pathological processes of multiple types of CVDs (Table 1).

**Table 1:** LLPS-Related Core Molecules/Complexes, Pathological Mechanisms and Associated CVDs

Disease type	Core molecules	Pathological mechanisms	Level	Ref.
MI	PGAM5-MAVS	Reduces PGAM5-MAVS co-condensation ⇢NLRP3 pyroptosis → alleviates MIRI	Vitro Vivo CS	[86]
	Ncl-HIF−1α	Ncl stabilizes HIF−1α mRNA via LLPS → upregulates NLRP3 → exacerbates myocardial pyroptosis	Vitro Vivo	[87]
	CBX7	Forms LLPS condensate → cuproptosis in aged hearts	Vitro	[88]
	VGLL3-EWSR1	Nuclear translocation + LLPS ⇢miR−29b → cardiac fibrosis	Vitro Vivo CR	[89]
Hypertension (Including Pre-eclampsia)	Alox5-Runx2	Promotes Runx2 nuclear LLPS → EGFR transcription → VSMCs hypertrophy	Vitro Vivo CS	[37]
	ZO−1	LLPS dysfunction → impairs tight junction → endothelial permeability	Vivo Spec	[91–94]
	G3BP1	G3BP1-mediated SG formation/dysfunction ⇢ Hedgehog/Wnt signal/oxidative stress → placental dysfunction + maternal endothelial injury → elevated sFlt−1/sEng → Preeclampsia	Vitro Spec	[90]
AS	BRD4-P300	ox-LDL → BRD4-P300 → LLPS forms super-enhancers → IL−6/TNF-α transcription	Vitro Vivo	[97, 98]
	Kindlin−2	Pulsatile shear maintains LLPS (endothelial protection); oscillatory shear inhibits it (increased permeability)	Vitro Vivo	[50]
	NLRP6	IDR-mediated LLPS → inflammasome assembly	Vitro Vivo	[103, 104]
	G3BP1-NLRP3	Autophagy-mediated LLPS → NLRP3 → exacerbates foam cell inflammation	Vitro	[34]
	DDR1-YWHAE	Flow-induced LLPS → forms condensate → YAP + pro-inflammatory genes	Vitro	[82]
	DDR1-LATS1	Mechanically-induced LLPS ⇢LATS1 → VSMCs phenotypic transformation + collagen deposition	Vitro	[19]
PH	LINC00599	Hypoxia → LINC00599 → m6A-mediated MYH9 LLPS → PASMC proliferation	Vitro Vivo CS CR	[70]
	ca-circSCN8A	Induces complex LLPS → stabilizes R-loop ⇢SLC7A11 transcription → PASMC ferroptosis	Vitro Vivo	[106]
	EphB4	Vascular extracellular matrix stiffening → EphB4 LLPS → sequestration of ANXA2 and YWHA → impairment of cytoplasmic retention of YAP → nuclear translocation of YAP → PASMC proliferation	Vitro Vivo	[107]
DbCM	YTHDF2	High glucose → Notch1 mRNA m6A → enhances YTHDF2 LLPS → accelerates mRNA degradation ⇢Notch1 pathway → mitochondrial fission abnormality + cardiac fibrosis	Vitro Vivo	[108–111]
HF	HIP−55	Phosphorylation defect ⇢LLPS ⇢P38/MAPK inhibition → aggravates myocardial apoptosis	Vitro Vivo	[31]
	JPH2	Oxidative stress promotes JPH2 nuclear LLPS + calpain−2 cleavage produces CTP → synergistically exacerbates myocardial remodeling	Vitro Vivo CS	[112–115]
DCM	LMNA Mutation	Disrupts lamellar structure → heterochromatin disorganization + LLPS aggregates	Spec	[121]
	RNP	Mutation impairs TNPO3 interaction → cytoplasmic mislocalization + harmful RNP LLPS → myocardial pathology → HF	Vitro Vivo CS	[124]
ORCM	DRP1	VD-mediated LLPS → cardiolipin binding + mitochondrial fission; high-fat diet disrupts LLPS ⇢mitophagy → myocardial mitochondrial disorders	Vitro CS	[128–130]
NIH	YAP	O-GlcNAcylation → YAP-STAT3 LLPS → nuclear translocation →CYR61/PCNA → VSMCs proliferation/migration	Vitro Vivo	[21]
SIMD	G3BP1	LPS → SGs LLPS ⇢TNF-α → mitochondrial membrane potential → alleviates contractile dysfunction; G3BP1 overexpression strengthens LLPS	Vitro	[134]
IR	SEMA6A	SEMA6A IDR-mediated LLPS → enriches RHOA/P300 → histone lactylation → PRMT5 → lactate-SEMA6A LLPS-PRMT5 positive feedback (accelerates abnormal angiogenesis)	Vitro Vivo	[135]
VM	DDX24	DDX24 IDR-mediated LLPS → regulates NPM1 phase behavior; E271K mutation impairs LLPS → disrupts nucleolar homeostasis + ribosome biogenesis → endothelial migration/tube formation	Vitro CS	[136]

⇢: Inhibition/Negative regulation; →: Leads to/Induces;

LLPS: Liquid-Liquid Phase Separation; CVDs: Cardiovascular Diseases; MI: Myocardial Infarction; MIRI: Myocardial Ischemia-Reperfusion Injury; VSMCs: Vascular Smooth Muscle Cells; AS: Atherosclerosis; ox-LDL: oxidized Low-Density Lipoprotein; PH: Pulmonary Hypertension; PASMC: Pulmonary Artery Smooth Muscle Cell; DbCM: Diabetic Cardiomyopathy; DCM: Dilated Cardiomyopathy; HF: Heart Failure; ORCM: Obesity-Related Cardiomyopathy; NIH: neointimal hyperplasianeointimal hyperplasia; SIMD: Sepsis-Induced Myocardial Dysfunction; IR: Ischemic Retinopathy; VM: Vascular Malformation; SGs: Stress Granules; RNP: Ribonucleoprotein; Vitro: In vitro only; Vivo: In vivo animal models; CS: Clinical samples; CR: Clinical correlation/clinical database evidence; Spec: Mechanistic speculation; Ref.: Reference

## Scientific hypotheses on irreversible phase transitions and conserved regulatory axes of LLPS in CVDs

Notably, the irreversible transition of BCs (e.g., liquid-to-gel/solid transformation, component solidification, or abnormal sequestration) may be a core driving factor of the irreversibility of pathological changes in end-stage CVDs. As an example, irreversible myocardial remodeling, defective phosphorylation of HIP-55 in HF triggers extensive LLPS, leading to the formation of solid-like aggregates that persistently inhibit the regulatory function of the P38/MAPK pathway [31]. Furthermore, coupled with the functional disorder of JPH2 nuclear condensates, including the formation of nuclear droplets by full-length JPH2 induced by oxidative stress and the nuclear localization of JPH2-CTP generated via calpain-2 cleavage, these events collectively promote cardiomyocyte apoptosis and adverse remodeling, ultimately resulting in irreversible cardiac dysfunction [112, 115]. Similarly, LLPS aggregates formed by LMNA mutants in dilated cardiomyopathy cause heterochromatin disorganization; these aggregates are difficult to reverse and continuously drive pathological myocardial remodeling [122]. This logic, whereby irreversible condensates lead to sustained abnormalities in core pathological pathways and ultimately induce permanent impairment of tissue function, may represent a key conserved mechanism underlying the irreversibility of end-stage CVDs.

**Fig. 4:**
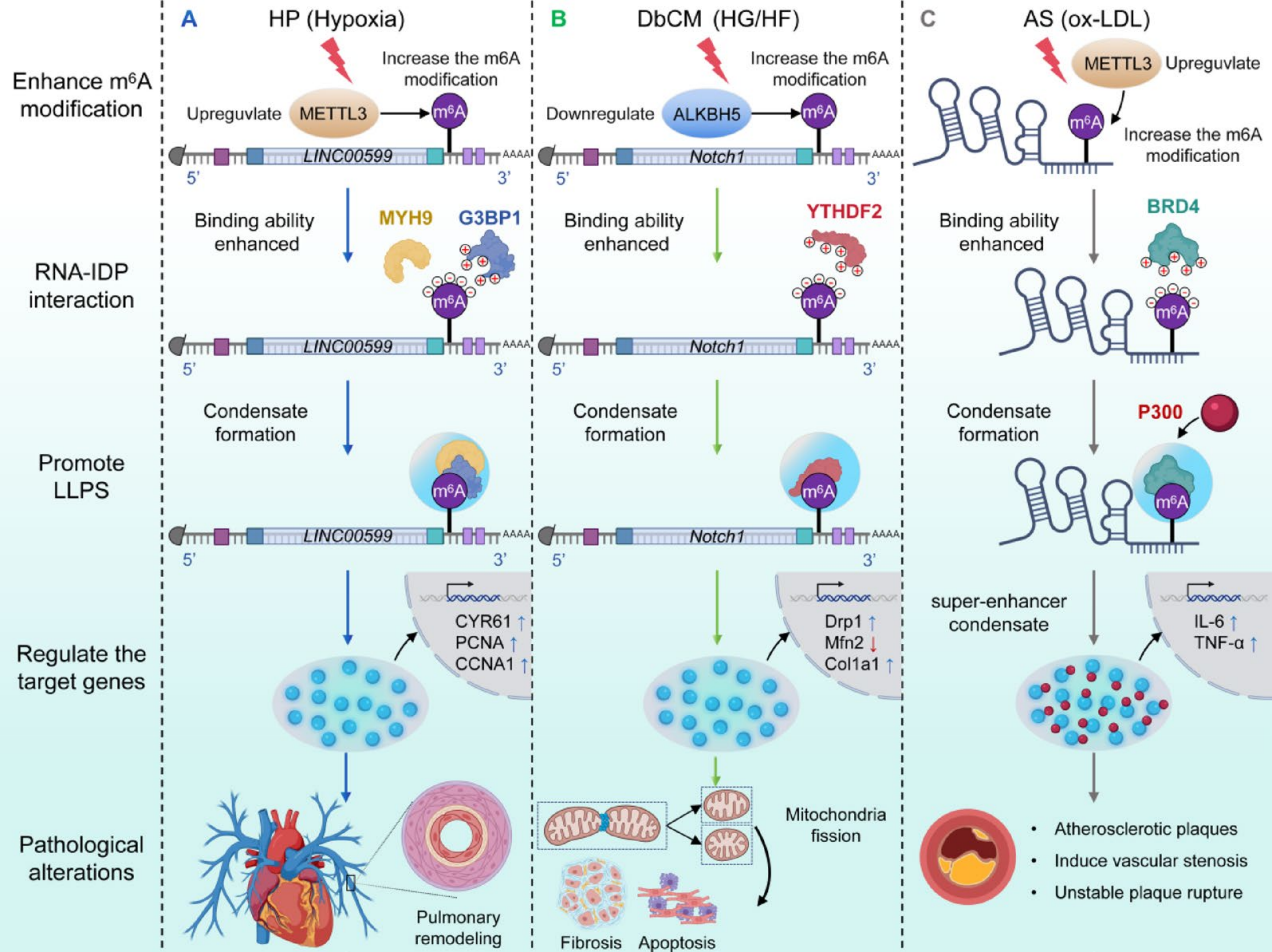
Molecular mechanisms of m6A modification-mediated RNA-IDP LLPS in regulating pathological processes of diverse CVDs. (**A**) PH (Blue arrows): Hypoxia induces the upregulated expression of METTL3, which elevates the m6A modification level of the long non-coding RNA LINC00599. This modification enhances LINC00599 binding to G3BP1/MYH9 and promotes LLPS condensate formation, thereby activating cell cycle-related genes such as CYR61, PCNA and CCNA1, and triggering pulmonary vascular remodeling. 1,6-hexanediol (1,6-HD) can disrupt these condensates and block the pathological progression. (**B**) DbCM (Green arrows): A high-glucose environment causes the downregulated expression of ALKBH5, leading to increased m6A modification of Notch1 mRNA. This modification strengthens the binding of Notch1 mRNA to YTHDF2 and facilitates LLPS condensate formation, accelerating Notch1 mRNA degradation. Inhibition of the Notch1 signaling pathway disrupts the expression of mitochondrial fission-related genes (upregulation of Drp1, downregulation of Mfn2) and fibrosis-related genes (e.g., Col1a1), ultimately resulting in myocardial fibrosis, cardiomyocyte apoptosis and abnormal mitochondrial fission. (**C**) AS (Gray arrows): Stimulation with ox-LDL induces the upregulated expression of METTL3, which increases the m6A modification of relevant RNAs. This promotes the formation of super-enhancer condensates between these RNAs and BRD4/P300, activates pro-inflammatory genes such as IL−6 and TNF-α, and drives atherosclerotic plaque formation, eventually leading to vascular stenosis and vulnerable plaque rupture. 1,6-HD can inhibit the function of these super-enhancer condensates

Although the regulatory role of LLPS in CVDs has been widely confirmed, existing studies have primarily focused on disease-specific, single-molecule local mechanisms. For instance, YTHDF2-mediated LLPS in DbCM depends on m6A modification of Notch1 mRNA [109], and m6A modification of LINC00599 promotes its co-condensation with G3BP1/MYH9 in PH ^*70*^. However, no study has systematically revealed the conserved regulatory rules of LLPS across different CVDs, hindering the development of cross-disease therapeutic strategies. Based on indirect evidence from existing literature, this review proposes an innovative hypothesis: m6A modification-mediated RNA-IDP LLPS might potentially act as a core conserved regulatory axis in CVDs. This hypothesis is currently supported by evidence from diabetic cardiomyopathy and PH, while its applicability to other CVDs remains to be further validated. At the molecular level, m6A modification increases the local negative charge density of RNA molecules, enabling stronger multivalent electrostatic interactions with arginine/lysine-rich positively charged regions of IDPs (e.g., YTHDF2, G3BP1). It also enhances hydrophobic interactions and π-π stacking, providing key driving forces for condensate formation [70, 110]. The specificity of different CVDs arises solely from differences in RNA-IDP combinations: Notch1 mRNA-YTHDF2 in DbCM [109], LINC00599 mRNA-G3BP1 in PH [70], and potentially m6A-dependent LLPS between lncRNA and BRD4 in AS [100], with highly consistent core regulatory logic (Fig. 4).

The proposal of this conserved axis breaks the limitations of disease-specific LLPS mechanism research and provides a novel target for developing broad-spectrum CVD therapeutic strategies. Targeting m6A modification-related enzymes (e.g., methyltransferase METTL3, demethylase ALKBH5) can regulate RNA’s LLPS potential at the source. Additionally, designing inhibitors against the m6A-binding domains of IDPs can specifically block the formation of pathological RNA-IDP condensates without the need for disease-specific optimization, significantly reducing clinical translation costs.

## Research progress of LLPS-targeted therapy for CVDs

As a core regulatory mechanism of CVDs, LLPS-targeted intervention strategies have emerged as a research focus, which mainly include four major directions: small-molecule regulation, biomaterial design, chiral intervention, and PTM-targeted regulation.

### Small-molecule LLPS Inhibitors/Modulators

Small molecules regulate LLPS by interfering with intermolecular interactions or altering the physical properties of condensates, which represents one of the strategies with the greatest clinical transformation potential at present:

#### 1,6-Hexanediol (1,6-HD)

1,6-Hexanediol (1,6-HD) is a classical and widely used mechanistic probe for LLPS research rather than a clinical candidate molecule. As a non-specific disruptor of hydrophobic interactions, it interferes with the structure of various BCs and is extensively employed to validate the LLPS dependence of biological processes in basic studies. In the cardiovascular system, 1,6-HD can effectively disperse the transcriptional condensates formed by BRD4 and MED1, thereby inhibiting the expression of genes associated with cardiac fibrosis [99, 137]. In addition, cyclin L1 (CCNL1) contains abundant IDRs, and studies have confirmed that it can undergo LLPS in cardiomyocyte nuclei. FRAP experiments showed that the highly dynamic EGFP-CCNL1 recovered approximately 65% of its fluorescence signal within 30 s after photobleaching, while 1,6-HD impaired the droplet fluidity of CCNL1, thereby promoting cardiomyocyte proliferation [138]. Meanwhile, 1,6-HD can block BRD4-MED1-dependent transcriptional LLPS in endothelial cells, inhibit the expression of cyclin A1 (CCNA1), arrest G1/S phase transition, and reduce angiogenesis capacity. After MI, timely revascularization or early establishment of coronary collateral circulation following chronic myocardial ischemia is a crucial strategy for repairing MI-induced injury. Studies have observed that 1,6-HD significantly inhibits angiogenesis with low toxicity to normal tissues in a mouse corneal neovascularization model, providing a novel potential strategy for the treatment of AS and tumor-associated vascular diseases [139].

#### Curcumin

Curcumin inhibits the LLPS of P300 and BRD4 as well as super-enhancer formation in foam cells by promoting the nuclear translocation of transcription factor EB (TFEB), thereby reducing inflammatory gene transcription. In an ApoE^−/−^ mouse model of AS, curcumin decreased lipid content and inflammatory cell infiltration in plaques, and this effect could be reversed by macrophage-specific BRD4 overexpression, confirming its anti-atherosclerotic effect through targeting LLPS [100].

#### Chrysophanol

Chrysophanol can inhibit the O-GlcNAcylation of YAP in VSMCs, reduce the LLPS activity of YAP and STAT3, and decrease YAP nuclear translocation. In a rat carotid artery ligation model, chrysophanol significantly inhibited neointimal thickness without obvious hepatorenal function impairment, serving as a candidate drug for the clinical treatment of NIH [21].

#### Resveratrol (RSVL)

RSVL can reduce the production of nucleic acid-induced type I interferons in cells by inhibiting G3BP1-mediated PS or disrupting G3BP1’s scaffolding function, thereby alleviating vascular inflammation associated with autoimmune diseases. This suggests a potential therapeutic strategy for inflammation-related vascular diseases such as AS ^*140*^.

#### Quercetin

Quercetin can dose-dependently inhibit the G3BP1/YWHAZ signaling axis, reduce cellular glycolysis and proliferation capacity, and indirectly affect LLPS-mediated vascular remodeling, indicating its application potential in the treatment of vascular NIH [141].

#### Therapy targeting the m6A-LLPS axis

As mentioned above, based on our hypothesis, the pathogenesis of CVDs might involve a conserved m6A-LLPS regulatory axisAs mentioned above, based on our hypothesis, the pathogenesis of CVDs might involve a conserved m6A-LLPS regulatory axis. In fact, the clinical translation of this potential therapeutic target has been preliminarily validated [110, 142]. Currently, drugs targeting non-coding RNAs (ncRNAs), such as ALN-PCSSC for LDL-C reduction and MRG-110/CDR132L for HF treatment, have entered clinical development [143, 144, 145]. Combined with regulators of m6A modification (e.g., ALKBH5 overexpression vectors), targeted enhancement of YTHDF-mediated LLPS regulation is expected further to improve the safety and efficacy of these therapeutic strategies.

### Biomaterial-based targeted LLPS regulation

By simulating the intracellular microenvironment or delivering functional molecules, biomaterials can precisely regulate the LLPS process and exhibit unique advantages in tissue repair for CVDs.

#### PS microfibrous hydrogels

By manipulating the LLPS process of gelatin and sodium hyaluronate, we constructed a microfiber network hydrogel with a microporous structure. This hydrogel can enrich laminin peptides and promote endothelial tube formation; when co-cultured with mesenchymal stem cells, it significantly enhances in vitro vascularization efficiency, thereby providing a novel scaffold material for tissue-engineering-based treatment of diseases such as MI and lower limb ischemia [146].

#### Multifunctional conductive hydrogels

Targeted interventions have been developed for LLPS-mediated pathological processes in MI. For example, as mentioned previously regarding CBX7, studies have identified δ-adrenosterone (δAe) as a selective inhibitor of CBX7. This compound can disrupt CBX7-ATP7A LLPS condensates, restore the membrane transport function and copper efflux activity of ATP7A, and alleviate caveolae-related mitochondrial damage in aged MI models. To enhance drug delivery efficiency, the research team designed a multifunctional conductive hydrogel with integrated antioxidative, proangiogenic, immunomodulatory, oxygen-releasing, and electroactive properties. A single injection enables the controlled release of δAe, combining the intrinsic reparative capacity of the hydrogel with the CBX7 inhibitory effect. This strategy significantly improved cardiac structure, function, and electrophysiological outcomes in aged mice and Bama minipig models of mitochondrial damage, demonstrating promising potential for clinical translation [89].

In addition, in MIRI, the weakly acidic microenvironment in the ischemic region is thought to be a key inducer of aberrant LLPS. Acidic conditions alter protein charge states, triggering pathological changes including abnormally increased SG formation and aberrant condensation of the mitochondrial fission protein DRP1. Combined with oxidative stress and electrical signaling disorders, these events form a network of multiple injuries. A recently developed injectable pH-responsive conductive hydrogel [147] does not explicitly mention or investigate LLPS in its original report. We therefore hypothesize, based on its pH-responsive and microenvironment-modulating characteristics, that this hydrogel may be compatible with the pathological features of aberrant LLPS in MIRI and could indirectly regulate pathological LLPS by delivering dual active factors in a microenvironment-responsive manner.

#### Injectable particulate hydrogels

An injectable granular hydrogel with a bicontinuous porous structure was fabricated by modulating the LLPS kinetics of the gelled phase and pore phase. In a murine cutaneous wound model, this hydrogel promoted M2 macrophage polarization and CD4⁺/Foxp3⁺ regulatory T-cell infiltration, accelerating angiogenesis and scarless wound healing. In a porcine skin defect model, its reparative efficacy outperformed that of the clinically used collagen/proteoglycan scaffold, providing a novel therapeutic strategy for the treatment of wound-healing disorders complicated by CVDs [148].

Notably, these findings were obtained from skin wound-healing systems rather than cardiovascular tissues. Based on its angiogenic and immunomodulatory properties, we propose this hydrogel as a potential translational strategy for wound-healing disorders complicated by CVDs, rather than an established LLPS-targeted intervention with validated efficacy and mechanisms in cardiac or vascular systems.

#### All-aqueous microfluidic systems

By mimicking the intracellular LLPS process, an all-aqueous emulsion microfluidic technology was developed to precisely fabricate uniformly sized MLOs mimics (e.g., SG-like droplets). This system can encapsulate cytokines such as vascular endothelial growth factor (VEGF). Regulating the LLPS-based release kinetics enables sustained VEGF delivery, promoting vascular regeneration and improving blood perfusion in a rat model of lower limb ischemia [149].

### Chiral nanomaterial-mediated LLPS intervention

Chiral nanomaterials specifically interfere with the LLPS process by regulating the chiral recognition and interaction of biomolecules, providing a new dimension for the precise treatment of CVDs.

#### Chiral ligand-modified nanoparticles

A library of gold nanoparticles modified with L- and D-cysteine was constructed. It was found that L-type nanoparticles promoted the LLPS assembly of SGs by enhancing the chiral recognition of G3BP1 protein, while D-type nanoparticles inhibited this process. In combined chemotherapy, L-type nanoparticles enhanced the LLPS of SGs in cardiomyocytes to protect normal cells from chemotherapy-induced damage, whereas D-type nanoparticles inhibited the LLPS of SGs in tumor cells to enhance chemotherapy sensitivity, achieving the dual effect of protecting normal tissues and enhancing therapeutic efficacy [150].

#### Chiral peptide-based condensates

As a speculative application and hypothetical translational direction, chiral peptides containing arginine-aromatic amino acid repeat sequences were designed, which can mediate LLPS through π-cation interactions to form condensates with adhesive properties. These condensates can mimic the glycine-rich protein (GRP), an IDP-rich protein in tick saliva, forming a gel-like sealing layer at the site of vascular injury to reduce bleeding and promote endothelial repair. In a mouse carotid artery injury model, chiral peptide condensates significantly shortened hemostasis time and promoted endothelialization of the injured blood vessel [151].

Importantly, this strategy is derived from tick adhesive biology and has not been directly tested in cardiovascular pathophysiological models. The mechanistic connection between tick-saliva-derived chiral peptides, LLPS mimicry, and cardiovascular endothelial repair remains speculative and warrants further experimental validation prior to potential clinical translation.

### Targeted therapy for PTMs

Targeted regulation of PTMs of LLPS serves as a core regulatory factor for LLPS, enabling precise intervention in LLPS functions.

#### Arginine methylation inhibitors

Inhibitors of PRMT5, such as EPZ015666, can suppress the arginine methylation of Kindlin-2 at residue R290, restore its LLPS activity, and promote the assembly of focal adhesions in endothelial cells. In an atherosclerotic model of ApoE^−/−^ mice, EPZ015666 reduced vascular endothelial permeability, diminished monocyte adhesion, and plaque formation. It exerted no significant toxicity on the hematopoietic system, thus providing a novel therapeutic target for the targeted treatment of AS [50].

#### O-GlcNAcylation Inhibitors

O-GlcNAc transferase (OGT) inhibitors (e.g., ST045849) can reduce the O-GlcNAcylation level of YAP, inhibit the LLPS and nuclear translocation of YAP and STAT3. In a rat carotid artery ligation model, ST045849 significantly inhibited NIH and downregulated the expression of proliferation-related genes such as CYR61 and PCNA, confirming its anti-NIH effect by targeting LLPS [21].

### Crosstalk between epigenetic modification and LLPS

In addition to classical PTMs, the crosstalk between epigenetic modification and LLPS provides a new dimension for therapy. DNA methylation can enhance the LLPS activity of MeCP2, which participates in transcriptional silencing by competing with histone H1 for chromatin binding [152]. Targeted DNA methylation inhibitors (e.g., decitabine) may improve AS by interfering with MeCP2-LLPS [153].

Furthermore, the histone methyltransferase EZH2 plays a role in AS [154], and its inhibitors can participate in related regulation by inhibiting EZH2-mediated LLPS to alleviate vascular inflammation. Histone deacetylase (HDAC) inhibitors (e.g., MS-275, Quisinostat A) regulate histone acetylation levels to interfere with the transcriptional regulatory function of LLPS condensates, thereby alleviating MIRI and protecting cardiac function after MI [155, 156].

## Challenges and prospects

### Current core challenges despite significant progress

Although remarkable advances have been made in the research of LLPS in CVDs, three core challenges remain to be addressed:

#### Mechanistic complexity

LLPS exhibits bidirectional roles in CVDs. For instance, LLPS of SGs protects cardiomyocytes during short-term ischemia but promotes cell apoptosis when sustained for a long period. However, the molecular threshold and regulatory network underlying this functional transition have not been fully elucidated [86]. Notably, dynamic m6A RNA modification, which is hypothesized to govern IDP LLPS in CVDs, is closely linked to this functional transition, though its relationship with pathological microenvironments and LLPS remains to be clarified.

#### Limitations of detection technologies

Current LLPS detection technologies face two core bottlenecks, which significantly hinder in-depth analysis of its dynamic regulatory mechanisms. On one hand, the ability for real-time dynamic monitoring in living cells is insufficient. Although existing techniques (e.g., FRAP, confocal microscopy) can achieve basic observation of LLPS condensates, they fail to simultaneously meet the requirements of high resolution and real-time performance. These methods cannot accurately capture transient changes in condensate formation/dissociation (e.g., millisecond-scale intermolecular exchange between phases) nor synchronously quantify microenvironmental characteristics (e.g., local viscosity, pH value, ion concentration), leading to incomplete interpretation of the regulatory network governing LLPS dynamic balance [157]. On the other hand, traditional labeling techniques have inherent interference issues: fluorescent tags (e.g., GFP-fused proteins, Atto series dyes) or chemical modifications often induce changes in protein conformation, loss of condensate liquidity (e.g., solidification), or even abnormal aggregation, which directly deviate from the physiological state of LLPS and introduce potential biases into experimental conclusions [158, 159, 160].

Novakovic et al. proposed the label-free LLPS detection technology REDIFINE in 2025 [161], using diffusion NMR to quantify droplet radius, diffusion coefficient, and interphase exchange without fluorescent labeling. While currently limited to in vitro systems, its multi-parameter, non-invasive approach overcomes limitations of traditional methods and is expected to enable quantitative detection of m6A-modified RNA–IDP condensates to validate our hypothesis in CVD cell models, addressing the technical gap in studying the m6A-LLPS axis in myocardial infarction, atherosclerosis, and PH.

#### Therapeutic safety

Small-molecule LLPS modulators (e.g., 1,6-hexanediol, 1,6-HD) may non-specifically interfere with physiological LLPS processes in normal cells (e.g., nucleolar assembly, DNA damage repair), resulting in off-target effects. Critical caveats: 1,6-HD displays prominent non-specific cytotoxicity and off-target effects, as it disrupts physiological LLPS processes in normal cells. These characteristics make it entirely unsuitable for clinical translation, and its application is limited exclusively to in vitro mechanistic validation and proof-of-concept studies rather than therapeutic use. Therefore, tissue/cell-specific LLPS-targeting strategies are urgently needed [162].

Currently developed small-molecule modulators of m6A enzymes (e.g., METTL3, ALKBH5) lack cardiovascular cell specificity; non-specific m6A regulation in cardiomyocytes, endothelial cells, or VSMCs may disrupt physiological RNA–IDP LLPS, causing impaired contractility, vascular barrier dysfunction, and other adverse cardiovascular effects.

Cancer research has confirmed that LLPS can regulate the biomineralization of gold nanocrystals within intracellular protein coronas, enabling fluorescent identification of tumors [163]. This idea may be translated to CVDs by designing LLPS-responsive nanocarriers for precise delivery of LLPS modulators such as 1,6-HD and curcumin, thereby increasing local drug concentrations and reducing systemic toxicity. This targeted delivery strategy could enable cell subtype-specific modulation of the m6A-LLPS axis in pathological cardiovascular cells (e.g., foam cells in atherosclerosis, hypertrophic VSMCs in hypertension) while minimizing effects on normal cardiovascular tissues.

### Promising directions for future research

Based on the current research status of LLPS in CVDs, future mechanistic investigations should not only address the challenge of mechanistic complexity (e.g., clarifying disease-specific LLPS regulatory networks and defining the molecular threshold and microenvironment-dependent relationships of LLPS functional transition) but also focus on breaking through core bottlenecks such as detection technology limitations and data modeling.

#### Development of high-resolution technologies for capturing dynamic LLPS processes

LLPS condensates exhibit highly dynamic, reversible, and MLOs. Traditional protein interaction identification methods (e.g., Co-IP, immunoprecipitation, conventional mass spectrometry) fail to capture their authentic composition and transient interactions accurately. The emergence of Proximity Labeling (PL) technology provides a powerful molecular tool for dissecting phase separated structures and core mechanisms. Its evolution toward higher spatial specificity and temporal control makes it more suitable for studying transient or aggregation-dependent interactions [164, 165, 166]. However, this technology still has limitations: for example, fusion of labeling enzymes (e.g., APEX2) may affect the function, localization, or interaction network of target proteins [167]. Therefore, future efforts could combine PL technology with fluorescent probes that sense microenvironmental properties (e.g., viscosity, pH) to achieve precise characterization of LLPS dynamic features under pathological conditions of CVDs.

#### Exploration of translational applications of artificial microbial MLOs

Building on artificial microbial MLOs, such as GGX motif IDR silk-like condensates and SUMOylation system condensates in *E. coli* [168, 169], these systems could be adapted to target CVD-related metabolites (e.g., lactate in myocardial ischemia, ox-LDL in atherosclerosis), offering novel intervention tools. Additionally, microbial MLOs engineered with functional domains of CVD-relevant m6A enzymes or m6A-binding proteins could specifically bind m6A-modified RNA in pathological cardiovascular cells, modulating abnormal RNA–IDP LLPS and enabling targeted regulation of the PTM-m6A-LLPS axis in major CVDs such as myocardial infarction and PH.

#### Development of IDR-independent LLPS prediction tools and pathogenic mutation localization models

Given the fragmentation and inconsistent standards of current LLPS databases, integrating PS data, disease-associated mutations, and drug-condensate interactions is essential to establish unified analytical criteria for dysregulated LLPS in CVDs. Most LLPS prediction tools (e.g., PhaSePred) focus on IDRs, yet non-IDR regions also mediate multivalent interactions [170]. To address this, PSPire integrates protein secondary structure and 3D conformation [171], while combining PSPHunter residue-level predictions with PTM data enables precise mapping of LLPS-regulating residues in CVD-related proteins [172]. Using dSCOPE, the impact of pathogenic mutations on spatial conformation, interaction potential, and PTM-dependent regulation of phase-separating regions can be systematically assessed [173], clarifying links between mutations, PTM signaling, and aberrant LLPS. This integrated model encompasses IDR and non-IDR modules and highlights PTM-mediated fine-tuning, offering a comprehensive, disease-targeted framework for investigating LLPS in CVDs.

#### Establishment of high-throughput screening and clinical translation systems for LLPS-targeted drugs in CVDs

For abnormal LLPS in CVDs, such as tight junction disruption in endothelial cells or YAP-STAT3 co-condensation in VSMCs, a drug screening platform based on Fluorescence Lifetime Imaging (FLIM) should be established for cardiovascular cells and ex vivo CVD models. Combined with condensate-modifiers therapies [174], a small-molecule library targeting CVD-relevant LLPS regulators, including scaffold protein phosphatases modulating YTHDF2 LLPS, can be designed. This library should also encompass modulators of key components of the PTM-m6A-LLPS axis, such as METTL3 in PH, ALKBH5 in diabetic cardiomyopathy, and m6A-binding proteins (YTHDF2, G3BP1) mediating RNA-IDP LLPS in myocardial infarction and atherosclerosis. Candidate drugs can be evaluated for their ability to restore LLPS condensate fluidity and function via FRAP and in vitro PS assays, with further validation in in vivo CVD models (e.g., ApoE^−/−^ atherosclerotic mice, myocardial infarction rats). For agents targeting the m6A-LLPS axis, a dual-validation system combining m6A modification assessment in cardiovascular tissues with LLPS functional analysis is essential to identify compounds that precisely restore physiological RNA-IDP LLPS and accelerate clinical translation of PTM-m6A-LLPS-targeted therapies for CVDs.

### Irreversible liquid-to-gel/solid transition of BCs as a Core mechanism in end-stage CVDs

The second scientific hypothesis proposed in this review is that irreversible phase transitions of BCs from dynamic liquid states to aberrant gel- or solid-like assemblies occur in end-stage CVDs and represent a unifying mechanistic basis underlying pathological irreversibility. This phase transition integrates multiple late-stage phenotypes, including refractory cardiac fibrosis, irreversible vascular remodeling, increased vascular stiffness, and therapy-resistant heart failure. Distinct from physiological, reversible LLPS, pathological liquid-to-gel/solid transitions sequester key regulatory molecules into nonfunctional aggregates, impair cellular compensatory mechanisms, and sustain pathological signaling.

Multiple lines of evidence indicate the widespread occurrence of irreversible BCs transitions across diverse CVDs. In heart failure, defective phosphorylation of HIP-55 leads to solid-like aggregates that persistently inhibit the P38/MAPK pathway, causing irreversible cardiomyocyte apoptosis [31]. In dilated cardiomyopathy, LMNA mutations induce the formation of stable lamin A/C aggregates, disrupting nuclear chromatin architecture and continuously driving myocardial remodeling [122]. In myocardial infarction, chronic ischemia promotes SG solidification, shifting their role from protective to pro-apoptotic signaling [86]. In atherosclerosis, hyperstabilized BRD4/P300 super-enhancer condensates sustain inflammatory gene expression, exacerbating plaque necrosis and vascular stiffening [100]. In diabetic cardiomyopathy, chronic hyperglycemia drives hardening of YTHDF2–RNA condensates, persistently suppressing cardioprotective signaling pathways [109].

The irreversible liquid-to-gel/solid transition is tightly regulated by multiple molecular determinants, including aberrant PTMs burden, abnormally elevated scaffold protein or RNA expression, condensate aging and hypermaturation, and oxidative stress or acidic pathological microenvironments. Together, these factors lower the gelation threshold, enhance excessive intermolecular crosslinking, and impede normal condensate disassembly [21, 31].

Importantly, irreversible BCs phase transitions in CVDs are mechanistically distinct from classical protein aggregation in neurodegenerative diseases (e.g., FUS and TDP-43 in amyotrophic lateral sclerosis, Tau in Alzheimer’s disease) [175]. In CVDs, these transitions are primarily characterized by loss of condensate dynamics, reduced molecular exchange, and functional sequestration of signaling hubs, rather than massive amyloid fibril formation.

Reversing or preventing pathological liquid-to-gel/solid BC transitions provides broad therapeutic potential in end-stage CVDs. Approaches include restoring PTM balance, promoting condensate disassembly, blocking aging, and targeting upstream triggers. This framework establishes a testable, mechanism-based hypothesis for next-generation targeted interventions.

### Research insights

- **What is currently known about this topic?**

1. LLPS mediates biomacromolecule condensation via non-covalent interactions, regulating cellular physiology and stress responses;
2. Dysregulated LLPS is linked to CVDs, involving IDPs/IDRs, nucleic acids, and PTMs as key drivers/regulators;
3. LLPS participates in pathological processes of common CVDs like MI, hypertension, Heart Failure, and AS.

- **What is the key research question?**

What conserved mechanisms underlie LLPS dysregulation in CVDs, and how to translate LLPS-targeted strategies into safe clinical applications?

- **What is new?**

1. Proposes a hypothesis that m6A modification-mediated RNA-IDP LLPS could act as a conserved regulatory axis in CVDs;
2. Identifies irreversible BC transitions as a pivotal driver of end-stage CVD pathological irreversibility;
3. Summarizes four novel LLPS-targeted therapeutic directions for CVDs.

- **How might this study influence clinical practice?**

Provides novel broad-spectrum therapeutic targets (e.g., m6A-RNA-IDP axis) and candidate interventions, advancing precision treatment for CVDs.

## Data Availability

No datasets were generated or analysed during the current study.

## Abbreviations

Alkbh5: AlkB Homolog 5
Alox5: Arachidonate 5-Lipoxygenase
APEX2: Ascorbate Peroxidase 2
AS: Atherosclerosis
ASC: Apoptosis-associated speck-like protein containing a CARD
ATG14: Autophagy-related protein 14
ATP7A: ATPase copper transporting Alpha
ca-circSCN8A: Circular RNA SCN8A with cytoplasmic accumulation
BCs: Biomolecular condensates
BRD4: Bromodomain-containing protein 4
CBX7: Chromobox protein homolog 7
CCNA1: Cyclin A1
CCNL1: Cyclin L1
circRNA: Circular RNA
CTD: Carboxy-terminal domain
CTGF: Connective tissue growth factor
CVDs: Cardiovascular diseases
CYR61: Cysteine-rich angiogenic inducer 61
DbCM: Diabetic cardiomyopathy
DCM: Dilated cardiomyopathy
DDR1: Discoidin domain receptor 1
DDX24: DEAD-Box Helicase 24
DRP1: Dynamin-related protein 1
EGFP: Enhanced green fluorescent protein
EGFR: Epidermal growth factor receptor
FISH: Fluorescence in situ hybridization
FLIM: Fluorescence lifetime imaging
EP300: E1A binding protein P300
EZH2: Enhancer of zeste homolog 2
FUS: Fused in sarcoma
EWSR1: Ewing sarcoma breakpoint region 1
FRAP: Fluorescence recovery after photobleaching
G3BP1: GTPase-activating protein binding protein 1
JMJD6: Jumonji domain-containing protein 6
JPH2: Junctophilin 2
HC: Heterochromatin
HDAC: Histone deacetylase
HF: Heart failure
HFD: High-fat diet
HIF-1α: Hypoxia-inducible factor-1α
HIP-55: Hematopoietic progenitor kinase 1 interacting protein 55
IDPs: Intrinsically disordered proteins
IDRs: Intrinsically disordered regions
IL-6: Interleukin-6
IR: Ischemic retinopathy
MIRI: Myocardial ischemia reperfusion injury
LATS1: Large tumor suppressor kinase 1
LCD: Low complexity domain
LINC00599: Long intergenic non-protein coding RNA 00599
LLPS: Liquid-liquid phase separation
LMNA: Lamin A/C
lncRNA: Long non-coding RNA
m6A: N6-methyladenosine
MAPK: Mitogen-activated protein kinase
MAVS: Mitochondrial antiviral signaling protein
MeCP2: Methyl-CpG binding protein 2
MLOs: Membraneless organelles
MI: Myocardial infarction
MOVLD: Multiorgan venolymphatic deficiency syndrome
MYH9: Myosin heavy chain 9
miRNA: MicroRNA
Ncl: Nucleolin
NIH: Neointimal Hyperplasia
NLRP3/6: NOD-like receptor family pyrin domain containing 3/6
NPM1: Nucleophosmin 1
OGT: O-GlcNAc transferase
ORCM: Obesity-related cardiomyopathy
ox-LDL: Oxidized low-density lipoprotein
PASMCs: Pulmonary artery smooth muscle cells
PCNA: Proliferating cell nuclear antigen
PERK: Protein kinase R-like endoplasmic reticulum kinase
PGAM5: Phosphoglycerate mutase 5
PH: Pulmonary hypertension
PIC: Pre-Initiation Complex
PL: Proximity labeling
RNA Pol Ⅱ: RNA polymerase Ⅱ
PRMT5: Protein arginine methyltransferase 5
PTMs: Post-translational modifications
PYD: Pyrin domain
RBM20: RNA binding motif protein 20
RBPs: RNA binding proteins
REDIFINE: Restricted difusion of invisible speciEs
RHOA: Ras homolog family member A
RNP: Ribonucleoprotein
RSVL: Resveratrol
Runx2: Runt-related transcription factor 2
SEMA6A: Semaphorin 6A
SGs: Stress granules
SIMD: Sepsis-induced myocardial dysfunction
SLC7A11: Solute carrier family 7 member 11
STAT3: Signal transducer and activator of transcription 3
STING: Stimulator of Interferon Genes
SUMO: Small ubiquitin-like modifier
TFEB: Transcription factor EB
TNF-α: Tumor necrosis factor-α
TNPO3: Transportin-3
TREX1: Three prime repair exonuclease 1
UTR: Untranslated region
VD: Variable domain
VEGF: Vascular endothelial growth factor
VGLL3: Vestigial like family member 3
VM: Vascular malformation
VSMCs: Vascular smooth muscle cells
YAP: Yes-associated protein
YTHDF2: YTH N6-methyladenosine RNA binding protein 2
YWHAE: Tyrosine 3-monooxygenase/tryptophan 5-monooxygenase activation protein ε
ZO-1: Zonula occludens-1
δAe: δ-Adrenalone
1,6-HD: 1,6-Hexanediol
